# Mental health problems among young people in substance abuse treatment in Sweden

**DOI:** 10.1186/s13011-020-00282-6

**Published:** 2020-06-24

**Authors:** Torkel Richert, Mats Anderberg, Mikael Dahlberg

**Affiliations:** 1 Department of Social Work, Malmö University, Citadellsvägen 7, 211 18 Malmö, Sweden; 2 Department of Pedagogy and Learning, Linnaeus University, Växjö, Sweden

**Keywords:** Young people, Adolescents, Mental health problems, Drug use problems, Substance abuse treatment, Gender differences

## Abstract

**Background:**

Young people with substance use problems face a high risk of co-occurring mental health problems, something that may involve a more difficult life situation, social problems as well as worse treatment outcomes. The aim of this study is to analyse self-reported mental health problems among young people receiving outpatient treatment for substance use problems in Sweden. We explore what types of mental health problems are more or less predominant, and whether there are significant differences between boys and girls. In addition, we analyse how various mental health problems covary with indicators of substance abuse severity.

**Methods:**

The study is based on structured interviews with 1970 young people enrolled at outpatient clinics in 11 Swedish cities. The data was analysed through frequency- and averages-calculations, Chi-square tests and multivariate logistic regression analyses.

**Results:**

Self-reported mental health problems were common among the young people in the study. A relatively large percentage of the total group (34–54%) reported problems such as concentration difficulties, sleeping difficulties, anxiety and depression. At the same time, many of the young people did not report any symptoms and only a small group, about 20%, reported diagnosed mental health disorders. The results show substantial gender differences, with girls reporting significantly higher levels of mental health problems.

Multivariate logistic regression analyses demonstrated significant associations between severity of drug use problems and anxiety, concentration difficulties, aggression, hallucinations and mental stress caused by experiences of trauma.

**Conclusions:**

Treatment needs are diverse within this group of young people who use drugs. Since girls report higher levels of all mental health problems, and a larger burden of psychosocial risk factors than boys, they are likely to require more comprehensive treatment interventions. The link between more severe drug problems and mental health problems points to the importance of exploring this relationship in treatment. A multidisciplinary approach, in which co-occurring problems can be addressed simultaneously, may be the best treatment form for many young people with drug problems.

## Introduction

The majority of young people with drug and alcohol problems suffer from concurrent mental health problems, referred to as co-occurring disorders or comorbidity [1–4]. Externalizing disorders such as Conduct Disorder (CD) and Attention Deficit Hyperactivity Disorder (ADHD) appear to be the most common mental health problems, but internalizing disorders such as depression, dysthymia and anxiety also commonly occur [5]. Boys are usually over-represented with respect to externalizing disorders, while the opposite holds true for internalizing disorders [6, 7], even though some studies report equal gender distribution [1, 5].

Most studies show that young people with co-occurring problems have more extensive drug problems, higher levels of social problems and criminality, as well as worse treatment outcomes and greater risk of relapse, compared with young people with substance use problems alone [1–4, 8–12].

On the other hand, there are also studies demonstrating that a relatively large proportion of adolescents with substance use problems do not report any mental disorders or symptoms at initiation of treatment [10, 13, 14], and studies showing minor differences between young people with and without mental health problems with respect to treatment involvement, dropout rate and treatment outcome [15–19].

Few Swedish studies have addressed the association between drug use and mental health problems among young people. This despite a recent trend with an increased prevalence of mental health problems among young people, and a development with a growing number of young people being diagnosed with, and treated for drug use problems [20, 21].

The Swedish studies that have been conducted – mainly based on adolescents in inpatient or emergency care – show that the vast majority of young people have mental health problems prior to onset of drug use, and that as many as 90% demonstrate co-occurring disorders [22]. There is little knowledge regarding mental health problems among broader groups of young people who use drugs in Sweden. There is also a lack of research on how different types of mental health problems may be linked to drug use severity among young people – this applies to Swedish research as well as to research on young people in general.

The aim of this article is to study self-reported occurrence of mental health problems among young people receiving outpatient treatment for substance use problems. We explore what types of mental health problems and social problems are more or less predominant, and whether there are significant differences in prevalence between boys and girls. In addition, we analyse how various mental health problems covary with indicators of substance abuse severity such as early onset of use, high frequency of use and poly drug use.

### Occurrence of mental health problems among young people in substance abuse treatment

As stated earlier, co-occurring mental health problems are relatively common among young people with drug and alcohol problems, even though such occurrence may demonstrate large variations [23, 24]. For example, several research reviews state that 50% to 90% of young people with drug and alcohol problems also have extensive mental health problems [1–3, 5, 12, 23–26].

Studies involving inpatient subjects generally demonstrate higher prevalence of mental health problems compared with studies on young people treated in outpatient or community-based programmes [1, 5, 7, 11, 27, 28]. In one Swedish study on young people enrolled at an emergency care center, 90% of girls and 81% of boys met the criteria for at least one psychiatric disorder in addition to their substance use problems [23]. Another Swedish study of young people receiving outpatient care for drug problems showed that 64% met the criteria for a psychiatric diagnosis [29]. An Australian study with a broad selection of young people in different types of treatment for substance use problems found that only one third had co-occurring mental health problems [14].

A research review by Couwenbergh and colleagues [5] concluded that externalizing disorders such as CD and ADHD are the most frequently found co-occurring psychiatric disorders among young people with substance use problems, followed by internalizing disorders such as depression or dysthymia and anxiety. However, the rate of various disorders varies greatly between different studies: 24% to 82% for CD, 3% to 48% for depression/dysthymia, 1% to 38% for anxiety disorders, 3% to 38% for ADHD and 10% to 12% for post-traumatic stress disorder (PTSD).

Several studies suggests that it is common for young people with drug and alcohol problems to have multiple co-occurring psychiatric diagnoses when in treatment [9, 11, 18, 30], and that the occurrence of problems varies with age, co-occurring problems being more common among people in their late teens or early adulthood [19, 31].

In summary, prior research points to great variation in levels of co-occurring mental health problems among young people in treatment for drug and alcohol problems, as well as what types of mental disorders occur most frequently. The variation found in the different studies may have several explanations. These include differences in selection criteria, types of care, assessment methodology, diagnostic tools, diagnostic systems and timeframes during which the conditions occur (e.g., the past month, past year, any time in life), which collectively make comparisons between different studies and contexts difficult.

### Associations between psychiatric disorders and severity of substance use problems

Only a few studies have investigated associations between various psychiatric disorders and indicators of severity of drug use problems among young people.

A study from Australia found that weakly or more frequent cannabis use in teenagers predicted an approximately twofold increase in risk for later depression and anxiety. In contrast, depression and anxiety in teenagers predicted neither later weekly nor daily cannabis use. Daily use in young women was associated with an over fivefold increase in the odds of reporting a state of depression and anxiety after adjustment for intercurrent use of other substances [32]. Similarly, a study on undergraduate students in the US found frequent marijuana use to be associated with depression and other substance use and alcohol-related negative outcomes [33].

Early onset of cannabis use has been associated with higher rates of later substance use, juvenile offending, mental health problems, unemployment and school dropout [34].

Results from a longitudinal study from substance use treatment facilities in Norway showed a co-occurrence between poly-drug use and mental distress. Mental distress increased both in magnitude and over time with the number of drugs used. The authors conclude that use of multiple drugs and mental distress appears strongly co-related over time [35].

There are various theories about the causal relationship between mental health problems and drug problems. According to several researchers, psychiatric problems usually precede drug and alcohol problems [12, 36–38]. This can for instance be explained by the drugs serving as a self-medicating function by alleviating stress and other unpleasant emotions [39]. However, the link between mental health problems and drug use cannot be explained solely on the basis of self-medication, since young people primarily state other reasons for using drugs [40–42]. The relationship between the two conditions can also be reversed, where drug and alcohol use increase the risk of mental health problems [32, 43, 44] . Another possible explanation is that underlying factors, such as difficult childhood circumstances or family problems may be the cause of both [4, 45, 46]. Mental health problems and substance abuse problems may also be intertwined and develop simultaneously over time. Finally, substance abuse and mental health problems may be mutually exclusive and coexist without any link [3, 32, 40]. It can be concluded that the association between drug and alcohol use problems and mental disorders is strong but extremely complex [25].

## Materials and methods

### Setting and sample

The study is based on structured interviews with young people receiving treatment for drug and alcohol problems. Data were gathered from specialized outpatient clinics for young people (13–21 years of age) with substance use problems in 11 Swedish cities. The participating cities are part of a researcher-practitioner collaboration network focusing on knowledge development in outpatient treatment for the present target group. The participating clinics represent Sweden’s three largest cities and eight additional cities around the country. The basic characteristics of the current sample are consistent with previous Swedish studies on the target group in outpatient treatment [13]. The centers, usually referred to as Maria clinics, are carried out as a collaboration between the social services and the healthcare system. All of the clinics offer various types of treatment for substance use problems, as well as counselling and support for young people and their families. Average length of care is 4–6 months. Services offered by all clinics include psychosocial and medical assessment with a focus on substance use problems, drug testing, individual or family counselling, and manual-based treatment programmes. Personnel include social workers, nurses, psychologists and doctors [13].

Between 2014 and 2016, 2099 young people begun outpatient treatment for substance abuse problems at the participating Maria clinics. All young people initiating treatment are asked to participate in an enrolment interview that is used for both treatment and research purposes. Of the total group, 129 individuals were excluded in the study because they did not want to participate or because the information gathered was incomplete. The study is based on a total of 1970 young people, of whom 27% were girls and 73% boys. The average age was 17.6 years for both boys and girls. 28% of the young people were either born outside of Sweden or have two parents born outside Sweden. This means that the sample, in this respect, is representative for young people in Sweden’s larger cities.

### Instruments and materials

UngDOK is a structured interview that was developed specifically for young people with various types of drug and alcohol problems. The primary purpose of the interview is to identify the problems, needs and current social situation of young people in order to arrive at the appropriate assessment, plan and implementation of treatment. The information gathered can also serve as a basis for follow-up and evaluation of the interventions and outcomes of the various local clinics. In a reliability and validation study, the quality of the UngDOK interview method was found to be satisfactory [47].

The interviews were conducted by the therapists at the time of admission. It contains a total of 75 questions covering the following ten aspects of life: housing and financial support, occupation, treatment history, criminality, childhood environment, exposure to violence, family and relationships, physical health, mental health, as well as alcohol and drug use.

The analysed variables describe individual characteristics regarding: social situation, drug and alcohol use, and mental health. The study only uses anonymized data, and permission for storage and processing of data for research purposes has been obtained from the research ethics committee at the National Board of Health and Welfare.

### Variables and statistical analysis

Initially, the study group was categorized by gender and the variables included in the study regarding mental health problems were analysed for gender differences. The data was processed and analysed by SPSS Statistics 24 software and Chi^2^ and t-tests were used for analysing levels of significance. To investigate the relationship between various mental health problems and drug use, multivariate logistic regression analysis with three models was carried out. As dependent variables, we used the following dichotomized variables that indicate severity of drug use: high frequency of use, i.e. two–three days a week or more for the primary drug (model 1), early onset of substance use, i.e. age 12 or younger for alcohol intoxication and age 13 or younger for drugs use (model 2), and presence of poly drug use, i.e. regular use of two or more drugs during the last three months (model 3). The independent variables in all three analysis models include age and gender, as well as self-reported mental health problems over the past 30 days: depression, anxiety, concentration difficulties, aggressive behaviour, suicidal thoughts, hallucinations, eating disorders, self-harming behaviour, and psychological stress from traumatic events (e.g. witnessed or being subjected to violence or sexual abuse). The results of these analyses are reported using odds ratios and significance levels. The explained variance for each model is presented using Cox & Snell and Nagelkerke.

## Results

Table 1 shows the childhood environment and social situation of the young people, as well as their self-reported drug and alcohol use at treatment initiation.

**Table 1:** Social situation and drug and alcohol use at time of admission (percentage distribution)

	Girls *N* = 541	Boys *N* = 1429	Total *N* = 1970	*p*
Living with parents	69	78	76	0.001*
Studies, practical placement or work	81	81	81	0.910
School problems	72	58	62	0.001*
Problems in childhood environment				
Financial problems	31	24	26	0.002*
Substance abuse	43	28	32	0.001*
Mental health problems	46	27	32	0.001*
Violence/assault	35	20	24	0.001*
Placement in foster care/residential home	19	17	18	0.078
Arrested by police	46	67	61	0.001*
Convicted of crime	22	39	34	0.001*
Victim of crime	50	46	47	0.110
Exposed to violence/abuse				
Physical	48	43	44	0,022*
Psychological	57	28	36	0.001*
Sexual	33	2	11	0.001*
Association with criminal/drug-abusing peers	66	65	65	0.655
Primary drug				
Alcohol	21	10	13	0.001*
Cannabis	69	85	80	0.001*
Other drugs	10	5	7	0.001*
Frequent substance use#	57	51	53	0.041*
Age at onset (years)	14.87	15.19	15.10	0.044*
AUDIT-C	53	38	43	0.001*
Poly drug use	28	24	25	0.058
Prior substance abuse treatment	28	25	26	0.344

*#Frequency of substance use 2–3 days/week or more*

*=p < 0.05

The majority of the young people live with their parents, are engaged in studies, traineeship, or work. However, most have or have had extensive difficulties in school that have impacted their attendance, satisfaction and performance. The percentage of girls with school problems is significantly greater than the percentages of boys.

About one third of the young people have grown up under difficult circumstances. Here too significant gender differences are found, where girls, to a much greater degree than boys, have experienced financial constraints, substance use problems, mental health problems and violence in their childhood environment.

A significant higher proportion of boys have been arrested by police or convicted of various crimes. Girls, however, report that they have been subjected to physical, mental and sexual violence or assault to a greater degree. An estimated two thirds of both boys and girls associate with friends who commit crimes or use drugs.

A number of gender differences were found regarding drug and alcohol use. For both genders, cannabis is the drug that causes the greatest problem or is the underlying reason for initiation of care, even though the percentage of boys that report this is significantly larger than the percentage of girls. The opposite gender pattern is seen with alcohol and other drugs. Girls initiate their primary drug somewhat earlier than boys and they also use that drug more often than boys. Gender differences are also reflected in alcohol use, where girls to a significant higher extent report risky alcohol consumption according to their AUDIT-C scores [48].

Table 2 shows that the most common self-reported mental health problems or disorders among young people over the past month are concentration difficulties, followed by sleeping difficulties, anxiety, depression, traumatic life events and problems controlling aggressive behaviour. The ranking is essentially similar for both boys and girls, although girls consistently report significantly higher levels.

**Table 2:** Self-reported mental health problems in the past 30 days (percentage distribution)

	Girls *N* = 541	Boys *N* = 1429	Total *N* = 1970	*P*
Mental health problems				
Sleeping difficulties	61	44	48	0.001*
Depression	49	28	34	0.001*
Anxiety	66	36	44	0.001*
Concentration difficulties	67	49	54	0.001*
Aggressive behaviour	25	17	19	0.001*
Suicidal thoughts	17	7	10	0.001*
Suicide attempts	3	1	2	0.003*
Hallucinations	8	4	5	0.001*
Eating disorders	12	4	6	0.001*
Self-harming behaviour	12	4	6	0.001*
Traumatic life event	44	26	31	0.001*
Medication for psychiatric disorders	31	14	19	0.001*
Neuropsychiatric diagnosis	19	22	21	0.164
Ongoing psychiatric care	32	17	21	0.001*

*=p < 0.05

Mental health problems such as suicidal thoughts, suicide attempts, hallucinations, eating disorders and self-harming behaviour are much less commonly reported (10% or less for the total group). These problems are however two to three times more common among the girls.

Almost half of the girls and a quarter of the boys have experienced a serious traumatic event or accident from which they have not yet psychologically recovered.

One-fifth of the young people state that they have an ongoing contact with psychiatry. About the same proportion state that they have received prescription medication for a psychiatric disorder or functional impairment and that they have a neuropsychiatric diagnosis. Girls report being in contact with psychiatric care or taking medication to a significantly larger extent, while no significant gender differences was found with respect to neuropsychiatric diagnosis.

Table 3 shows how frequency of drug use, age at onset of drug use, and occurrence of poly drug use covary with nine self-perceived mental health problems, as well as with age and gender.

**Table 3:** Self-reported mental health problems in the past 30 days in relation to high frequency of drug use, early onset of drug use and poly drug use (controlling for age and gender)

	Frequency of drug use (*N* = 1776)			Age at onset of drug use (*N* = 1757)			Poly drug use (*N* = 1813)		
	OR	*P*-value	95% CI	OR	*P*-value	95% CI	OR	*P*-value	95% CI
Constant	0.008	0.001		1.214	0.751		0.002	0.001	
Age	1.264	0.001***	1.206–1.324	0.857	0.001***	0.802–0.915	1.296	0.001***	1.235–1.359
Gender	0.879	0.297	0.690–1.120	0.993	0.967	0.722–1.366	0.857	0.254	0.657–1.118
Depression	1.312	0.050	1.000–1.722	1.047	0.808	0.723–1.516	1.250	0.138	0.931–1.667
Anxiety	1.719	0.001***	1.318–2.241	1.412	0.068	0.975–2.045	1.358	0.048*	1.002–1.839
Concentration difficulties	1.408	0.003**	1.124–1.765	1.555	0.008**	1.122–2.155	1.434	0.007**	1.106–1.860
Aggressive behaviour	1.381	0.020*	1.052–1.812	1.576	0.007**	1.130–2.199	1.147	0.359	0.856–1.539
Suicidal thoughts	1.160	0.463	0.781–1.721	0.650	0.115	0.381–1.111	0.962	0.847	0.650–1.424
Hallucinations	0.849	0.501	0.526–1.368	1.601	0.099	0.915–2.803	1.652	0.041*	1.021–2.674
Eating disorders	1.549	0.054	0.993–2.419	1.304	0.318	0.774–2.197	1.146	0.545	0.737–1.783
Self-harming behaviour	0.880	0.599	0.546–1.418	0.601	0.849	0.460–1.567	1.102	0.692	0.682–1.778
Traumatic life event	0.986	0.910	0.775–1.255	1.335	0.075	0.971–1.836	1.302	0.043*	1.008–1.683
Cox & Snell	0.151			0.037			0.135		
Nagelkerke	0.202			0.068			0.199		

* = < 0.05 ** = < 0.01 *** = < 0.001

Young people with a high frequency of drug use (two–three days a week or more) were significantly more likely to report anxiety (OR = 1.719), concentration difficulties (OR = 1.408), and difficulty in controlling aggressive behaviour (OR = 1.381), in comparison with young people with a low frequency of drug use. High frequent users were also significantly more likely to be older (OR = 1.264).

Young people with early onset of drug use were significantly more likely to report concentration difficulties (OR = 1.555), and difficulty in controlling aggressive behaviour (OR = 1.576), in comparison with those not reporting early onset of drug use. People with an early onset of drug use were significantly less likely to be older (OR = 0.857).

Young people with poly drug use were significantly more likely to report anxiety (OR = 1.358), concentration difficulties (OR = 1.434), hallucinations (OR = 1.652) and traumatic life events (OR = 1.302) in comparison with those not reporting poly drug use. People with poly drug use were also significantly more likely to be older (OR = 1.296).

## Discussion

The aim of this study was to analyse self-reported mental health problems among young people receiving outpatient treatment for substance use problems in Sweden. We explore what types of mental health problems are more or less predominant, how various mental health problems covary with indicators of substance abuse severity, as well as gender differences related to drug use and mental health problems.

The study shows that the young people who initiate outpatient treatment at Maria clinics for drug and alcohol problems are characterized by both heterogeneity and pronounced differences between boys and girls. Overall, the most common self-reported mental health problems among the young people are concentration difficulties, followed by sleep disturbances, anxiety, depression, traumatic experiences and difficulties managing anger and violent behaviour. This ranking is largely in line with many other studies [5]. Although the list of problems is ranked similarly by gender, the percentage of girls with various mental health problems is consistently higher than boys, sometimes many times higher.

A relatively large percentage (34–54%) of the total group report self-assessed problems such as depression, anxiety and concentration difficulties. These findings are in line with current descriptions of health among Swedish young people in general, where a growing number of boys and girls report psychosomatic symptoms and stress-related mental health problems [20]. The increase in mental health problems encompasses broad groups of young people in Sweden, which indicate that the underlying causes are located in living conditions and environments that are common to most young people. The Public Health Agency of Sweden [20] has highlighted inadequacies in the school system and the increasing demands for performance that many young people experience, as well as concerns about entering into adult life and the future demands of the labor market as the primary factors underlying this trend. The fact that young people spend more time on social media has also been highlighted as a possible risk factor. Another possible explanation is that the social stigma of mental illness has decreased, something that can lead to more young people reflecting on and reporting mental health problems, as well as seeking help for these problems to a higher extent [20]. Social norms that place a premium on success and perfection, when combined with a weak social position in relation to peers, has also been shown to generate stress-related mental health problems among young people in upper secondary school [49].

A smaller but still significant group of the young people (about 20–30%), report problems that may indicate more serious mental health and drug use problems. These include, among other things, being exposed to a serious traumatic event, having a diagnosed neuropsychiatric disorder, being in psychiatric treatment, taking prescription medications for specific mental health problems, having a poly drug use, and prior experience of substance abuse treatment. A small percentage of the young people (2–10%) reported problems such as hallucinations, eating disorders, self-harming behaviour, suicidal thoughts and suicide attempts, which may indicate severe or long-lasting psychiatric conditions.

However, the majority of the young people (about 70–80%) do not show signs of having severe mental health problems. Many of these young people are from relatively stable home environments and are well-integrated in society with a satisfactory school career, regular leisure activities and positive relationships with their parents [13]. They have less severe alcohol and drug problems which likely stem from more experimental or social drug use motivated by curiosity or sporadic use with friends [50]. The findings of this study, in this respect, contradict several earlier studies that concluded that it is the rule rather than the exception that young people with drug and alcohol problems have co-occurring mental health problems [1–3, 5, 12, 22]. This difference can be explained in part by the different target groups that have been studied: young people in outpatient care tend to be a more heterogeneous group, while those recruited from inpatient care or emergency care usually have more extensive social and mental health problems [1, 5, 7, 11, 27].

What specific mental health problems are related to more severe substance use problems? The study results suggest a strong link between concentration difficulties and severity of drug use problems. High frequency of use, early onset of drug use, as well as poly drug use, were associated with higher likelihood of reporting concentration difficulties. Concentration difficulties may be a cause of early onset of drug use for some adolescents and a consequence of extensive drug use for others. According to our findings, anxiety problems appear to be clearly linked with poly drug use and frequent drug use, but not to early onset of use. Anxiety problems in adolescence have been shown to predict alcohol use problems in young adulthood [46]. Regarding cannabis, the opposite appears to be true: cannabis use in adolescence can increase the risk of anxiety and depression later in life [32, 46, 51]. Aggressive behaviour, which may be linked to the condition often referred to as conduct disorder, was mainly associated with early onset of drug use. An association also suggested by several other studies [5, 22, 45]. In our study, hallucinations and psychological stress from traumatic events – which may be indications of more severe mental health problems – are only linked to poly drug use. According to a study by Harrison, Fulkerson & Beebe [52], young people with experiences of physical and sexual abuse reported high rates of poly drug use, initiated substance use earlier than their peers and gave more reasons for using, including use to cope with painful emotions. PTSD (post-traumatic stress disorder), which can be a result of traumatic experiences such as abuse, has also been shown to increase the risk of developing substance use problems [46, 53]. Furthermore, results from a longitudinal growth research study from substance use treatment facilities in Norway show that use of multiple drugs and mental distress are strongly co-related over time [35].

Several mental health problems such as depression, suicidal thoughts, eating disorders and self-harming behaviour did not show significant associations with either high frequency of use, early onset of drug use, or poly drug use. This finding suggests that young people with substance use problems may have co-occurring mental health problems without any clear link between the two [25]. However, these mental health problems are more common in the study group compared with young people in general [20]. This may in part be explained by the fact that the young people in the study, compared with young people in the general population at large, have more difficult childhood circumstances and a greater social vulnerability.

The explained variance in the three analysed models (Table 3) is relatively low (according to Cox & Snell and Nagelkerke), which suggests that a number of other factors are in play. One possible explanation is that a large number of social factors that could affect the relation between drug use and mental health are not included in the model, such as social relationships, social norms, peer pressure, school problems, and access to drugs.

The study findings show substantial gender differences, with a larger percentage of girls reporting both mental health problems and receiving psychiatric care and prescription medications for psychiatric disorders. The gender differences – with the exception of neuropsychiatric diagnoses – are significant for all reported conditions. The results are in line with several other studies on young drug users [14, 54–56]. Clear gender differences in mental health problems are also found in Swedish young people in general. The proportion of young people reporting symptoms of mental health problems in Sweden has doubled over the past two decades. This trend is true for both boys and girls, but the prevalence is about twice as high for young girls [20]. Regarding drug problems, the differences between girls and boys have decreased in Sweden in recent decades. However, gender differences remain as the proportion of girls with drug problems is smaller than the proportion of boys, while girls generally report more severe drug problems [13].

Experiences of traumatic events, violence and physical abuse, which are more common among the girls in the study, can be one explanation for their higher level of mental health problems. It is also probable that some girls begin to use drugs or alcohol to alleviate or manage such painful experiences and the stress that they cause [57–60]. The girls in the study generally show somewhat earlier onset of drug use and more frequent use. However, the multivariate analyses did not point to any distinct gender differences, suggesting that the link between severity of substance use problems and various mental health problems holds regardless of gender.

There are some limitations to the study. The empirical material is based on self-reported data gathered from interviews at initiation of treatment. It is difficult to draw conclusions about causal relationships between mental health problems and substance use problems with this type of cross-sectional data. Consequently, the study is not based on established psychiatric diagnoses. However, the reliability of the screening instruments and assessment methodology in identifying and assessing mental health problems in children and young people is also generally weak, which may result in both under-reporting [4], and over-reporting of mental health problems [61]. Diagnoses are often re-classified, their criteria and boundaries change over time, and they are affected by societal developments [40, 62].

### Implications

Studying self-reported mental health problems in young people with drug and alcohol problems can yield important new knowledge. Greater knowledge of how various mental health, drug-related and social problems covary, can be of general value and may also be instrumental in developing and organising treatment interventions for the target group. Staff and managers at the Maria clinics can benefit from data about their target group at an aggregated level. This can provide guidance on which problems and combinations of problems are more or less common and thus what types of treatment efforts may need to be strengthened or prioritized. It is not unusual for individuals with both mental health problems and drug and alcohol problems to be passed from one care provider to the next without receiving relevant help for their co-occurring problems [5]. A multidisciplinary approach is far preferable, in which both conditions are addressed and concomitant treatment goals are formulated together with the young person. Integrated treatment for co-occurring problems has strong scientific support [2]. In this regard the Maria clinics represent a positive example of working with the target group, in which treatment for both conditions can be provided within one and the same service, such as psychosocial treatment combined with medication [25]. However, there is a risk that an increased focus on medical conditions may contribute to a psychiatrization and medicalization leading to the subordination of social issues in substance abuse treatment [63]. It is of great importance that these young people are also provided with the opportunity for social inclusion and support for meaningful employment and recreational activities to help them address both their drug use problems and mental health issues. This is obvious given that the majority of young people in the study have school problems and socialize with friends who commit crimes or use drugs.

Early onset of drug, frequent use and poly drug use were all significantly associated with increased likelihood of reporting various mental health problems, in particular anxiety, concentration difficulties and problems controlling aggressive behaviour. Poly drug use was also linked to experiences of hallucinations and traumatic life events. This points to the importance of screening for mental health problems when treating young people with drug use problems. It also points to the importance of exploring the specific roles or functions that drug use have for young people, especially how these functions may relate to traumatic events and different mental health problems.

Since girls have a larger burden of psychosocial risk factors than boys, they are also more likely to require more comprehensive and multidimensional treatment interventions that extend over a longer period [28, 56]. It is especially important to consider difficult home environments and reported severe psychological problems from which many young people suffer. Past traumatic experiences also need to be considered and addressed in treatment, especially among girls, who are more likely to have had such experiences [56, 64, 65]. Since a large proportion of girls have previously been in psychiatric care, it should also be possible to identify such girls in order to offer more relevant support at an earlier stage.

## Conclusions

In summary, the study shows that various forms of self-reported mental health problems are common among young people with drug and alcohol problems who begin outpatient treatment in Sweden. At the same time, many young people report that they do not have any symptoms and only a small percentage report documented psychiatric conditions. The need for support or treatment for mental health problems within this group is thus highly diverse, but should be analysed and assessed on a case-by-case basis. Since girls report higher levels of all mental health problems, and a larger burden of psychosocial risk factors than boys, they are likely to require more comprehensive treatment interventions. The link between the severity of drug problems and mental health problems points to the importance of highlighting and exploring this relationship when treating young people and specifically the role played by the drug use. A multidisciplinary approach, in which co-occurring problems can be addressed simultaneously may be the best treatment form for many young people with drug problems.

Additional studies are needed in this field. There is a need for studies that investigate different causal relationships between mental health problems and the severity of drug use problems. There is also a need for qualitative studies that focus on young people’s different motives for and experiences of drug use, and how these relate to mental health problems.

## Data Availability

The datasets used during the current study are available from the corresponding author on reasonable request.

## Abbreviations

ADHD: Attention Deficit Hyperactivity Disorder
AUDIT-C: The Alcohol Use Disorders Identification Test – Consumption
CD: Conduct Disorder
PTSD: Post-traumatic stress disorder
SPSS: Statistical Package for the Social Sciences
